# Cost-effectiveness analysis of fruquintinib for metastatic colorectal cancer third-line treatment in China

**DOI:** 10.1186/s12885-020-07486-w

**Published:** 2020-10-13

**Authors:** Zhi Peng, Xingduo Hou, Yangmu Huang, Tong Xie, Xinyang Hua

**Affiliations:** 1 Department of Gastrointestinal Oncology, Key Laboratory of Carcinogenesis and Translational Research (Ministry of Education/Beijing), Peking University Cancer Hospital and Institute, No.52, Fusheng Road, Haidian District, Beijing, 100037 China; 2 School of Public Health, Peking University, No.38, Xueyuan Road, Haidian District, Beijing, 100191 China; 3 Department of Global Health, School of Public Health, Peking University, No.38, Xueyuan Road, Haidian District, Beijing, 100191 China; 4 Nuffield Department of Population Health, University of Oxford, Old Road Campus, Headington, Oxford, OX3 7LF UK

**Keywords:** Metastatic colorectal cancer, Cost-effectiveness analysis, Fruquintinib

## Abstract

**Background:**

In this study, we analyze the cost-effectiveness of fruquintinib as third-line treatment for patients with metastatic colorectal cancer in China, especially after a recent price drop suggested by the National Healthcare Security Administration.

**Methods:**

A Markov model was developed to investigate the cost-effectiveness of fruquintinib compared to placebo among patients with metastatic colorectal cancer. Effectiveness was measured in quality-adjusted life years (QALY). The Chinese healthcare payer’s perspective was considered with a lifetime horizon, including direct medical cost (2019 US dollars [USD]). A willing-to-pay threshold was set at USD 27,130/QALY, which is three times the gross domestic product (GDP) per capita. We examined the robustness of the model in one-way and probabilistic sensitivity analysis.

**Results:**

Fruquintinib was associated with better health outcomes than placebo (0.640 vs 0.478 QALYs) with a higher cost (USD 20750.9 vs USD 12042.2), resulting in an incremental cost-effectiveness ratio (ICER) of USD 53508.7 per QALY. This ICER is 25% lower than the one calculated before the price drop (USD 70952.6 per QALY).

**Conclusion:**

After the price negotiation, the drug becomes cheaper and the ICER is lower, but the drug is still not cost effective under the standard of 3 times GDP willing-to-pay threshold. For patients with metastatic colorectal cancer in China, fruquintinib is not a cost-effective option under the current circumstances in China.

## Background

With increasing incidence and mortality, colorectal cancer is a major public health problem, and has brought constantly heavy economic burden. Over 1.8 million new colorectal cancer cases and 881,000 deaths were estimated to occur in 2018, ranking third in incidence and second in mortality all over the world [1]. The statistics from China not only showed a high prevalence of colorectal cancer in this country with 376,300 new cases and 191,000 deaths in 2015, but a steadily increasing trend of incidence and mortality rates over the years which added great stress on Chinese public health [2–4].

Systematic antineoplastic agents represent the main approach in the management of metastatic colorectal cancer (mCRC). Fluorouracil, oxaliplatin, irinotecan, bevacizumab, cetuximab, and panitumumab are widely used alone or in combination for the treatment [5–10]. However, progression was still common after the initial and subsequent palliative care [11]. Effective third line or later line treatment is urgently needed to release the medical and economic pressure in China.

Fruquintinib is an effective small molecular vascular endothelial growth factor receptor (VEGFR) inhibitor. In the absence of a randomized clinical trial (RCT) comparing fruquintinib with any of the two other agents approved in this setting, TAS-102 and regorafenib, indirectly demonstrated little differences in efficacy, if any, among the three drugs [12]. In a phase III multicenter RCT, the FRESCO (fruquintinib efficacy and safety in 3+ line colorectal cancer patients) trial, fruquintinib demonstrated the effectiveness in prolonging the progression-free survival (PFS) (3.7 versus 1.8 months; HR 0.26, 95% CI, 0.21–0.34) and overall survival (OS) (9.3 vs 6.6 months; HR 0.65, 95% CI, 0.51–0.83) in metastatic colorectal cancer patients compared with placebo. Moreover, fruquintinib was subsequently approved by Chinese National Medical Products Administration for metastatic colorectal cancer refractory to standard regimen. However, according to the trial data, 61.2% of the patients experienced grade 3 or grade 4 adverse effects (AEs) in the fruquintinib arm comparing with 19.7% in the placebo arm. The most frequently reported grade 3 or grade 4 adverse effects with fruquintinib were hypertension (21.2%), hand-foot skin reaction (10.8%), and proteinuria (3.2%) [13].

Unlike regorafenib, TAS-102 or other imported anti-angiogenesis drugs, fruquintinib is produced in China, which might lead to lower pricing and higher accessibility in mCRC patients in China. In the end of 2019, China started a price negotiation process, and decided to include fruquintinib in the medical insurance by 2020, in the hope of decreasing out-of-pocket payment for patients, as well as supporting domestic origin drugs. As a result, the price for fruquintinib has decreased by over 60%, and the out-of-pocket payment has decreased from over CNY (Chinese yuan) 20,000 to CNY 2381 per month after medical insurance. However, whether fruquintinib earns incremental benefit is unknown. There is no previously reported cost-effectiveness data on fruquintinib. Therefore, in this study based on the survival benefits reported in the FRESCO trial, we built a Markov model to investigate the cost-effectiveness of fruquintinib, compared to supportive care among patients with metastatic colorectal cancer who had previously progressed after at least 2 lines of chemotherapy in China.

## Methods

### Model structure

The Markov is a commonly used approach in health economics evaluation. It comprises a finite set of health states in which an individual can be found and analyzes uncertain disease process over time through cycles consisting of short time intervals, in which individuals can move between states or remain in the same state. In this study, a Markov model was constructed using the TreeAge Pro 2011 software (https://www.treeage.com) to simulate the mCRC disease process. Statistical analyses were performed in R software (http://www.r-project.org).

**Fig. 1:**
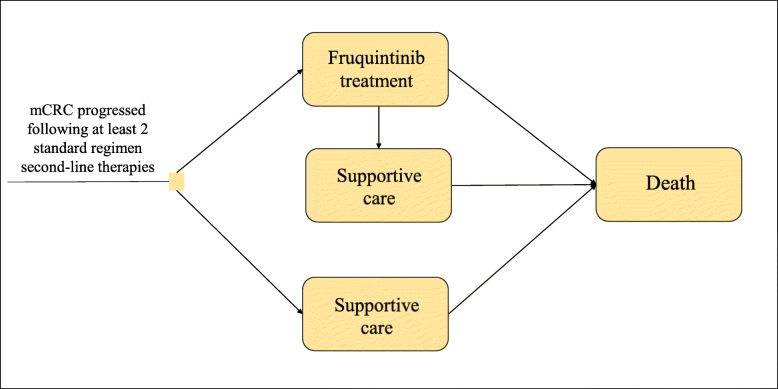
Markov model. mCRC, metastatic colorectal cancer

As shown in Fig. 1, all patients initially entered the model in one of the two initial states, which included treatment with fruquintinib or best supportive care (BSC). At the end of each model cycle, patients could remain in the original state or move to other states. Treatment with fruquintinib was discontinued in patients who experienced disease progression, grade 3/4 adverse events (AE) or intolerance to treatment. Patients discontinuing treatment were assigned to receive BSC. All patients in each state could experience progression to death.

Mortality rates of each group were calculated based on the survival data derived from the FRESCO trial [11]. Weibull model was used to fit the OS curves published in the FRESCO trial. The mortality rates were calculated based on the equation ${P}_{(t)}=1-\frac{S\left(t+1\right)}{S(t)}$, where *P*_(*t*)_ and *S*_(*t*)_ represented mortality rates and survival rates at cycle t, respectively. Based on the median treatment duration published in the FRESCO trial, treatment discontinuation probabilities in the fruquintinib group were estimated supposing they were exponentially distributed. For the mortality and treatment discontinuation risk beyond the follow-up time in the trials, we estimated them based on the fitted survival models. In order to prove that the OS curve generated by the Markov model simulation had a close approximation comparing with those shown in the FRESCO trial, we performed internal model verification.

A lifetime horizon was used in the Markov model. Each cycle in the model lasts for 4 weeks, which includes a 3-week daily fruquintinib treatment routine and a 1-week break. The outputs of the model are cost and QALY. QALY is a composite measure of quality of life and quantity of life, which typically assigns to each period of time a weight corresponding to the health-related quality of life during that period. The incremental cost-effectiveness ratio (ICER) was calculated as the difference in cost between the two treatments arms divided by the difference in QALYs.

### Cost estimates

In the study, we considered direct medical costs, which related to costs for drugs, BSC, as well as treatments for grade 3/4 AEs. Societal costs like travel fees and time costs were not taken into consideration. All the parameters were stated in 2019 US dollars.

In order to estimate drug price for each unit, we considered two different situations. Fruquintinib was newly added into the drug lists of Chinese medical insurance in 2020 with a lower price after the negotiation by the National Healthcare Security Administration. After the negotiation, the price of two types of fruquintinib are USD 384.7 / box (5 mg / capsule), and USD 288.5/ box (1 mg / capsule). The recommended dose for fruquintinib is 5 mg per day with a unit cost of USD 53.8/5 mg.

We also calculated the cost-effectiveness before the price negotiation, which refers to the price of fruquintinib in the Chinese drug market which takes drug donation activities from the fruquintinib manufacturers. Before the negotiation, the prices of fruquintinib were USD 1064.3/box (5 mg/capsule) and USD 892/box (1 mg/capsule). The unit cost was USD 162.3. Considering the drug donation activities, in the first, second and fifth cycles of fruquintinib treatment, patients payed for the drug themselves; in other cycles of treatment, the drug was handed to the patients free of charge.

Some grade 3/4 AEs were included in the model, whose incidence was significantly different between two groups in the FRESCO trial, including hand-foot syndrome, hypertension, diarrhea, proteinuria and platelet count decreased. Costs for treatments for grade 3/4 AEs and best supportive care were estimated based on guidelines and articles recently published [14–17].

### Utility estimates

To calculate QALYs in the model, we adjusted survival time by utility. There was no quality-of-life data collected in the FRESCO trial, therefore, each health state was assigned with a health utility score based on literature [14]. Referring to the trial, the value assigned to the basic utility for all patients was 0.66, and we used 0.735 and 0.59 as the upper and lower boundaries of utility in the sensitivity analysis.

In addition, the duration-adjusted decreases in utility caused by grade 3/4 AEs were subtracted from the baseline utility. The disutility of AEs was estimated based on published manuscripts [14, 15]. The unmeasured decreases for the same health states in the FRESCO study were expected to be similar. Duration of AEs was estimated based on clinical experience. Hand-foot syndrome was assumed to be with a disutility of − 0.116 and a duration of 14 days, diarrhea was assumed to be with a disutility of − 0.103 and a duration of 5 days, platelet count decreased and hypertension were assumed to be with a disutility of 0. The annual discounting of the costs and utility in this analysis was set at a rate of 3%.

### Sensitivity analysis

Internal model validations were performed, to demonstrate that the OS curves generated by the Markov model simulation closely approximated those presented in the FRESCO trial.

We performed one-way sensitivity analysis and probabilistic sensitivity analysis in order to quantify uncertainty and evaluate the robustness of the model. In one-way sensitivity analysis, the parameters analyzed were varied within ±20% of their baseline values. The baseline values, ranges, and distributions used in the analysis are listed in Table 1. In probabilistic sensitivity analysis, a Monte Carlo simulation was performed for 1000 times, each time randomly sampling from all the parameters of their distributions. Cost-effectiveness acceptability curves were used to demonstrate the uncertainty on the result in relation to possible values of the willingness-to-pay (WTP) thresholds.

**Table 1:** Baseline values, ranges, and distributions for sensitivity analysis

Variable	Value	Range	Distribution
AEs with fruquintinib			
Hypertension Hand-foot syndrome Diarrhea Platelet count decreased	0.212 0.108 0.029 0.025	0.170 to 0.254 0.086 to 0.130 0.0232 to 0.0348 0.02 to 0.03	Beta Beta Beta Beta
AEs with best supportive care			
Hypertension	0.022	0.0176 to 0.0264	Beta
Cost of best supportive care	1415.4	1022.8 to 2021.5	Gamma
Cost of fruquintinib after price negotiation	1128.8	903.0 to 1354.5	Gamma
Cost of fruquintinib before price negotiation	3408.5	2726.8 to 4090.2	Gamma
AE cost, USD			
Hypertension Hand-foot syndrome Diarrhea Platelet count decreased	59.1 134.48 44.3 3551.7	47.28 to 70.92 107.58 to 161.38 28.5 to 54.6 3156.8 to 3980.2	Gamma Gamma Gamma Gamma
Utility	0.66	0.59 to 0.735	Beta
AE duration, days			
Hand-foot syndrome Diarrhea	14 5	11.2 to 16.8 4 to 6	Gamma Gamma
AE disutility			
Hand-foot syndrome Diarrhea	−0.116 −0.103	−0.139 to − 0.093 − 0.123 to − 0.082	Beta Beta
Discount rate	0.03	0.00 to 0.05	

According to the World Health Organization (WHO) Guide to Generalized Cost-Effectiveness Analysis [18], the WTP threshold in the cost-effectiveness acceptability curves was set at USD 27,130/QALY, which is three times the per capita Chinese GDP of China in 2018.

### Scenario analysis

In order to compare the effect of medical insurance, we made a scenario analysis with the total price before price negotiation to see how medical insurance affects the overall cost effectiveness of fruquintinib.

## Results

### Base case results

As shown in Table 2, after price negotiation, the estimated cost for the fruquintinib group is USD 20750.9, which is higher than the USD 12042.2 estimated for the placebo group, resulting an incremental cost (IC) of USD 8708.7. Compared with the placebo group, the fruquintinib group gains 0.163 QALYs. The ICER of the study is USD 53508.7 per QALY.

**Table 2:** Base case model results after price negotiation

Strategy	Fruquintinib	Placebo
Cost, USD	20,750.9	12,042.2
Effect, QALY	0.6404	0.4776
IC, USD	8708.7	–
IE, QALY	0.16275	–
ICER, USD per QALY	53,508.7	–

*Abbreviations*: *IC* incremental cost, *IE* incremental effectiveness, *ICER* incremental cost-effectiveness ratio, *QALY* quality-adjusted life-years

### Sensitivity analyses

**Fig. 2:**
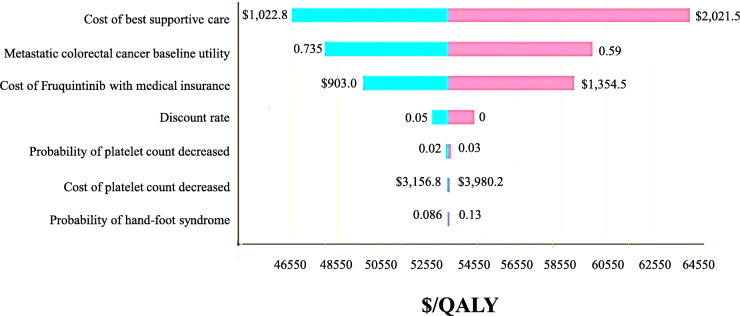
Invariable sensitivity analyses after price negotiation

Tornado diagrams are used to present the results of one-way sensitivity analysis (Fig. 2). The cost of best supportive care, the cost of fruquintinib, and the baseline utility value have the largest impact on the ICER. The parameters with less influence on the ICER are the duration and disutility for AEs. In the range of variation of each parameter, the ICER remains >USD 27,130/QALY.

**Fig. 3:**
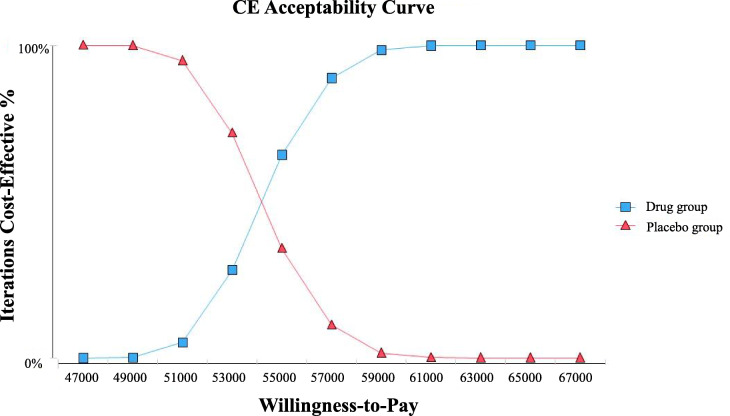
Cost-effectiveness Acceptability Curve after price negotiation

The cost-effectiveness acceptability curves are shown as Fig. 3. As the value of WTP, the curves demonstrate the probability of fruquintinib to be cost-effective. These curves show that, after price negotiation, the cost-effective possibility of fruquintinib is close to 0% at a WTP value < USD 49,000 per QALY. When the WTP value is about USD 54000 per QALY, There is a 50% probability that fruquintinib is cost-effective.

### Scenario analysis

As shown in Table 3, before the price negotiation, the estimated cost for the fruquintinib group increases into USD 23590.0, the ICER of the study is USD 70952.6 per QALY.

**Table 3:** Base case model results before price negotiation

Strategy	Fruquintinib	Placebo
Cost, USD	23,590.0	12,042.2
Effect, QALY	0.6404	0.4776
IC, USD	11,547.8	–
IE, QALY	0.16275	–
ICER, USD per QALY	70,952.6	–

*Abbreviations*: *IC* incremental cost, *IE* incremental effectiveness, *ICER* incremental cost-effectiveness ratio, *QALY* quality-adjusted life-years

## Discussion

This study primarily investigated the cost-effectiveness of fruquintinib as third-line treatment for mCRC. Fruquintinib, as a potent and highly selective small molecular inhibitor targeting VEGFR-1, VEGFR-2 and VEGFR-3, significantly prolonged the PFS and OS in a phase III RCT [13]. In our study, fruquintinib was associated with relatively longer QALYs (0.64, vs 0.48 with BSC). However, the cost of fruquintinib is expensive, and the ICER remained nearing USD 53508.7 per QALY after adjusting sensitive variables, which was far higher than the acceptance threshold set at three-fold the per capita Chinese GDP. Thus, the results suggested an unlikelihood of incremental benefit of fruquintinib in the mCRC third-line treatment in China.

Standard regimens containing fluorouracil, oxaliplatin, irinotecan, and targeted agents interfering with the epidermal growth factor receptor (EGFR), vascular endothelial growth factor (VEGF) pathways are widely used in the first-line and second-line treatment for mCRC [5–7]. However, few drugs except regorafenib and TAS-102 were approved in the mCRC third-line settings by Food and Drug Administration (FDA) [19, 20]. In addition, the drugs are not without great cost. Considering the survival benefit, the quality of life and the expensive price, the cost-effectiveness of mCRC third-line treatment drugs is uncertain.

Recently, the Chinese government has included fruquintinib in the medical insurance, in the hope of decreasing the drug price, as well as supporting domestic origin drugs. After the price negotiation, both the total price of the drug, and the ICER decreased. Before being included in the medical insurance, the cost of fruquintinib was a heavy burden for most cancer patients’ families. After entering the medical insurance, the official announced price reduced more than 60%, with a reimbursement proportion of 70%. This greatly reduces the financial burden on the patient’s family, allowing more patients to fruquintinib for a long time, thereby extending survival. However, our results indicated that fruquintinib is still unlikely to be cost-effective.

This is similar with the cost-effectiveness study on TAS-102 and regorafenib in the United States, where both were not cost-effective at standard WTP thresholds (ie, USD 150,000 per QALY) relative to BSC, and showed no clear evidence for better relative value [21]. These findings were not surprising, since many researchers have argued that the price for cancer drugs was independent of novelty, that the pricing models were not rational but simply reflected what the market will bear [22, 23]. Researchers have suggested that policy makers should evaluate the effects of pricing policies on both affordability and access to anticancer drugs, as well as on the anticancer drug-development pipeline [23]. However, in the case of China, the non-cost-effectiveness of fruquintinib might also be related to the thresholds commonly used in cost-effectiveness studies, where the limited GDP owing to the developing status of China might result in lower thresholds. Several researchers suggested that the use of conventional cost-effectiveness analysis thresholds recommended by World Health Organization might cause misleading conclusions, especially for low- and middle-income countries with low GDP [24, 25]. Patients WTP and pricing policy from government should find a new benchmark for cost-effective care.

Based on data from the CORRECT trial, Goldstein et al. reported an additional 0.04 QALYs (0.13 life years) at a cost of USD 40,000 and ICER of USD 900,000 per QALY for regorafenib. Regorafenib was demonstrated to be of minimal incremental benefit at high costs per QALY in the United States [14]. Additionally, Cho et al. revealed similar results, showing that BSC was more cost-effective than regorafenib at standard WTP [26]. On the other hand, although TAS-102 provided an additional 0.041 QALY compared with regorafenib, it showed the same negative result of TAS-102 for cost-effectiveness analysis in the United States [26]. Another comparative cost-effectiveness study conducted in England and Wales, showed that TAS-102 was associated with longer life year (LY) (0.92 versus 0.82) and QALY (0.82 versus 0.51) when compared with regorafenib, which was consistent with the United States showing the TAS-102 superiority. The ICER for TAS-102 was £51,194 per QALY. After the probabilistic analysis, TAS-102 showed an > 60% probability to achieve a 3-months OS gain [27]. Similar to regorafenib, fruquintinib failed to show cost-effectiveness in our study. And although TAS-102 luckily achieved potential benefit in England and Wales, the worldwide cost-effectiveness of the drug is controversial. The results jointly provided useful references to assess the cost of mCRC third-line treatment, however, due to the policy and price variations, it is challenging to compare the cost-effectiveness of the drugs across different countries. The most cost-effective choice of mCRC third-line treatment in China needs further investigation.

Anti-angiogenesis agents play a pivotal role in the mCRC treatment. Aside from regorafenib and fruquintinib, adding biochemical targeting VEGF pathway, like bevacizumab, aflibercept and ramucirumab, also significantly prolonged the PFS and OS in the first-line and second-line mCRC treatment [28–31]. By pooling data from six RCTs, Hurwitz et al. demonstrated the prolongation of PFS (9.1 versus 6.9 months; HR, 0.58; 95% CI, 0.46–0.73; *p* < 0.0001) and OS (19.8 versus 17.6 months; HR, 0.81; 95% CI, 0.70–0.93; *p* = 0.0034) by adding bevacizumab to chemotherapy backbone in the mCRC first-line treatment. However, the cost-effectiveness study revealed that bevacizumab gained minimal survival benefit at USD 571,240 per QALY in the first-line treatment. Moreover, in the second-line treatment, bevacizumab failed to show cost-effectiveness, with ICER USD 364,083 per QALY. Aflibercept combined with FOLFIRI regimen (folinic acid, luorouracil and irinotecan) on the other hand, prolonged 2.23 months PFS and 1.44 months OS in a phase III RCT, after model adjustment the ICER turned out to be > £62,000 per QALY (USD 95,000 per QALY), which was thought to be not cost-effective in the United Kingdom either [32]. To date, all the three anti-angiogenesis drugs were thought to have similar efficacy and survival benefit in the second-line mCRC treatment when added to FOLFIRI. However, ramucirumab was far more expensive than the other two agents [33]. Although no studies have demonstrated the cost-effectiveness of ramucirumab to date, the results may probably be negative too. So far, all of the approved anti-angiogenesis drugs in the mCRC treatment failed to show cost-effectiveness, no matter in which line. However, owing to the lack of anti-angiogenesis drugs cost-effectiveness study in China, adding these drugs to mCRC patients’ treatment plan may need a careful discussion between physicians and the patients.

On the other hand, adding cetuximab, an anti-EGFR monoclonal antibody, to chemotherapy also showed prolongation of survival, with an ICER of USD 2.9 million per LY. Interestingly, by testing KRAS mutation status as a stratification strategy, the ICER was reduced to USD 2.8 million per LY [34]. Using biomarker to select patients that may benefit can be an effective way to improve the cost-effectiveness of fruquintinib. However, as there are no currently validated biomarkers for anti-angiogenesis agents efficacy in mCRC, further studies in this field are urgently needed.

There are several limitations in our study. First, this study was not prospective, and drug price and costs were assumed as static. AEs were given pre-defined durations, which may not fully reflect the real world. Secondly, we used conventional WHO’s cost-effectiveness thresholds, however, the threshold might not be the best option for oncology drugs, thus the conclusion of not cost-effectiveness should be drawn with caution [35, 36]. Lastly, all the results were based on the FRESCO study, a multicenter RCT conducted only in China, which might not be the case for other countries.

## Conclusion

In conclusion, this is the first study to investigate the cost-effectiveness of fruquintinib in the mCRC third-line treatment. With the ICER far exceeding the three-fold Chinese per capita GDP, fruquintinib seems unable to be cost-effective in China under current standard. Finding dependable biomarkers for fruquintinib, establishing new value-based pricing and payment system are needed in the future.

## Data Availability

All data analysed during this study are included in published article.

## Abbreviations

QALY: Quality-adjusted life years
USD: US dollars
ICER: Incremental results effectiveness ratio
GDP: Gross domestic product
mCRC: Metastatic colorectal cancer
VEGFR: Vascular endothelial growth factor receptor
PFS: Progression-free survival
RCT: Randomized controlled trial
OS: Overall survival
AE: Adverse effect
CNY: Chinese yuan
FRESCO: Fruquintinib efficacy and safety in 3+ line colorectal cancer patients
BSC: Best supportive care
WTP: Willingness-to-pay
WHO: World Health Organization
IC: Incremental cost
IE: Incremental effectiveness
EGFR: Epidermal growth factor receptor
VEGF: Vascular endothelial growth factor
FDA: Food and Drug Administration
CORRECT: Colorectal cancer treated with regorafenibor placebo after failure of standard therapy
LY: Life year
FOLFIRI: Folinic acid, luorouracil and irinotecan
